# Supplementary material for: Learning curve and surgical outcome of robotic assisted colorectal surgery with ERAS program
Source: Sci Rep. 2022 Nov 29;12:20566. doi: 10.1038/s41598-022-24665-w (PMC9709162; doi:10.1038/s41598-022-24665-w)
Supplement: Supplementary file 2 — Supplementary Information 2. [file 41598_2022_24665_MOESM2_ESM.zip › RAL Raw data-Ñ[▒K/054/HS2_ñw╝╨░O▒Kñσ.pdf]

正式摘要(電子簽章：000000209964748)	【讀取】憑證	
W52 / W52 ☐英文標題(注意:無英文姓名-請補病人基本檔!)		

臺中榮民總醫院

出院病歷摘要

轉入號： 轉出號：

[redacted]

(8)流水編號	
(9)入院日期	110/01/31 (2021) 直腸外科 W52 - 155
(10)轉科日期	
(11)出院日期	110/02/08 (2021) 住院天數計 8 日

診斷

- 入院：1. Diverticulosis of colon, s/p Robot Assisted Colectomy on 2021/01/20
  2. Cyst over right upper liver (16cm), s/p liver Laparoscopic fenestration for hepatic cyst on 2021/01/20
  3. Hypertension, under medication
  4. Appendicitis , s/p appendetomy in 1961
  5. Uterus prolapse, s/p hysterectomy in 2010.
  6. Rupture of right knee ligament, s/p repair surgery
- 出院：1. Post op ileus
  2. Diverticulosis of colon, s/p Robot Assisted Colectomy on 2021/01/20
  3. Cyst over right upper liver (16cm), s/p liver Laparoscopic fenestration for hepatic cyst on 2021/01/20
  4. Hypertension, under medication
  5. Appendicitis , s/p appendetomy in 1961
  6. Uterus prolapse, s/p hysterectomy in 2010.
  7. Rupture of right knee ligament, s/p repair surgery

主訴

- Vomitting once this morning.

病史

- The 80 year old female had history of
  1. Diverticulosis of colon, s/p Robot Assisted Colectomy on 2021/01/20
  2. Cyst over right upper liver (16cm), s/p liver Laparoscopic fenestration for hepatic cyst on 2021/01/20
  3. Hypertension, under medication
  4. Appendicitis , s/p appendetomy in 1961
  5. Uterus prolapse, s/p hysterectomy in 2010.
  6. Rupture of right knee ligament, s/p repair surgery
  She just discharged from our CRS ward on 1/27. Poor appetite and abdominal distension were noted at home. Vomitting once at 1/30 morning, then she was brought to our ER. Epigastric pain was told and she denied fever, dysuria. Stool and gas passage (+). Abdominal CT revealed segmental dilatation of jejunum with focal wall thickening, adhesional obstruction with ischemia change (Ser2, #58) can't be R/O. Under the impression of post op ileus, she was admitted for further survey and treatment. NPO, NG decompression, ABx of MDZ were given at ER.

體檢發現

- 9/28 CT: diverticulum. huge liver cyst. 16 cm.
  - General appearance :
    - easy looking , conscious clear , without cardiopulmonary distress
    - Vital signs自動帶入date 體溫(BT) 脈搏(P) 呼吸(R) 血壓(BP) 身高(BH) 體重(BW) BMI
      20210131 36.4℃ 79次/分 18次/分 168/68mmHg 155.8cm 50.5kg 20.8
    - Vital signs: B.P. 168/68 mmHg , P.R. 79 /min , R.R. 18 /min , B.T. 36.4 ℃
  - HEENT :
    - Eyes: no anemic conjunctiva, no icteric sclera, normal EOM
    - ENT: no otorrhea, no rhinorrhea, no erythematous mucosa, no injected tonsil, no deformity
  - Neck: supple, freely movable, trachea midline,no deformity, no engorged jugular vein , no palpable thyroid or lymph node enlargement
  - Chest:
    - Inspection: Grossly normal, symmetric and fully expansion, no accessory muscle used, no operation scar, no spider angiomata, no gynecomastia
    - Palpation: no palpable mass or nodule, no subcutaneous emphysema, no tenderness
    - Ausculation: clear breathing sound
  - Heart:
    - Percussion: normal heart size
    - Ausculation: regular heart beat, normal S1, S2, no gallop, no murmur
  - Abdomen:
    - Inspection: in
      - flat contour, symetrically, no scar, no stria, no dilated veins, no rashes
    - Ausculation: normoactive bowel sound, no abdominal bruits

- palpation: soft, no muscle guarding or rigidity, no local tenderness, no rebounding pain, no palpable mass, no palpable pulsation
  - Percussion: no shifting dulllness, no hepatomegaly, no splenomagaly
-

Peripheral pulsation :	CA	BA	RA	FA	PA	PTA	DPA
Right side :	++	++	++	++	++	++	++/+++
Left side :	++	++	++	++	++	++	++/+++

- Genito-anus: no tenderness or palpable mass lesion or rectal shelf, normal external genitalia, normal anal tone, brown stool

CRS CONDITION

- Imaging studies(Sono , CT of abdomen and chest Films):
  - Segmental dilatation of jejunum with focal wall thickening, adhesional obstruction with ischemia change (Ser2, #58) can't be R/O.
-
  - Lung

手術日期及方法

住院治療經過

- After admission, NPO, NG decompression, ABx of MDZ were given. She was tolerated to try water since 2/1, and we removed the NG on 2/2. We also adjust hypertension medication at hospital course and let her try BLAND NO.I DIET since 2/4. Due to she tolerate BLAND I DIET well, we shifted intake to BLAND II DIET. She can be discharge under stable condition and tolerate BLAND II diet.

合併症

一般檢查

- 累積報告 :

DATE	TIME	ALB	TP	BIL.T	BIL.D	ALKP	AST	ALT	LDH	CHOL	TG	HDL-C	LDL	UA	NH3	AMY
1100130	1441			0.52	0.12	81	29	26								92
1100201	1145									139	127					
1100204	0905	3.2		0.23	0.10		21	8								

DATE	TIME	WBC	RBC	HGB	HCT	MCV	PLT	BLST	NEUT	BAND	SEG	LYM	MON	EOS	BAS
1100130	1439	12430		13.8			355		83.0			11.3	5.3	0.2	0.2
1100204	0918	8160	3.96	12.3	38.5	97.2	323		77.7			11.9	7.8	2.2	0.4

DATE	TIME	NA	K	CL	CA	BUN	CREAT	Mg	P	CRP	ESR	PCT	LIPASE	LACTATE
1100130	1441	140	3.4			21	0.84						52	
1100130	1612									1.387				
1100201	1145							2.2	2.5					
1100204	0905	143	3.6		7.9	11	0.58							

特殊檢查

病理報告

其它

出院時情況

- 3.改門診治療

出院指示

藥名	劑量	劑量單位	用法	頻次	數量	數量單位	現況	天數	單位
Through tab	1	TAB	PO	QN	14	TAB	TH	14	天
Gaslan tab 40mg	1	TAB	PO	QID	56	TAB	TH	14	天

出院計劃

主治醫師 2222H 林俊余 / 住院醫師 2353C 楊立宇

正式摘要(電子簽章：**000000209964748)**

W52 / W52

doctorRank:B
